# Strategies for ocular siRNA delivery: Potential and limitations of non-viral nanocarriers

**DOI:** 10.1186/1754-1611-6-7

**Published:** 2012-06-11

**Authors:** Ajit Thakur, Scott Fitzpatrick, Abeyat Zaman, Kapilan Kugathasan, Ben Muirhead, Gonzalo Hortelano, Heather Sheardown

**Affiliations:** 1 Institute of Biomaterials and Biomedical Engineering, University of Toronto, Toronto, ON, Canada; 2 School of Biomedical Engineering, McMaster University, Hamilton, ON, L8N 3Z5, Canada; 3 Faculty of Medicine, University of Manitoba, Winnipeg, MB, Canada; 4 Faculty of Medicine, University of Toronto, Toronto, ON, Canada; 5 Department of Pathology & Molecular Medicine, McMaster University, Hamilton, ON, L8N 3Z5, Canada; 6 Department of Chemical Engineering, McMaster University, Hamilton, ON, L8N 3Z5, Canada

**Keywords:** Biomaterials, siRNA, Drug delivery, Endosomal escape, Nanocarriers, Ocular siRNA delivery, RNAi

## Abstract

Controlling gene expression via small interfering RNA (siRNA) has opened the doors to a plethora of therapeutic possibilities, with many currently in the pipelines of drug development for various ocular diseases. Despite the potential of siRNA technologies, barriers to intracellular delivery significantly limit their clinical efficacy. However, recent progress in the field of drug delivery strongly suggests that targeted manipulation of gene expression via siRNA delivered through nanocarriers can have an enormous impact on improving therapeutic outcomes for ophthalmic applications. Particularly, synthetic nanocarriers have demonstrated their suitability as a customizable multifunctional platform for the targeted intracellular delivery of siRNA and other hydrophilic and hydrophobic drugs in ocular applications. We predict that synthetic nanocarriers will simultaneously increase drug bioavailability, while reducing side effects and the need for repeated intraocular injections. This review will discuss the recent advances in ocular siRNA delivery via non-viral nanocarriers and the potential and limitations of various strategies for the development of a ‘universal’ siRNA delivery system for clinical applications.

## Introduction

### Challenges of posterior segment ophthalmic therapeutics

**Figure 1:**
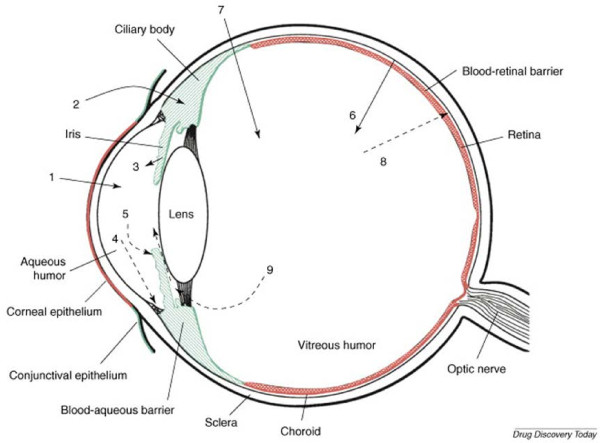
**Schematic representation of the various routes of ocular drug delivery and drug elimination from the eye. 1)** trans-corneal permeation, **2)** non-corneal drug permeation, **3)** drug delivery to the anterior chamber via the BAB, **4)** drug elimination from the anterior chamber via the trabecular meshwork and Sclemm’s canal, **5)** drug elimination from the anterior chamber into the uveoscleral circulation, **6)** drug delivery to the posterior chamber via the BRB, **7)** intravitreal drug delivery, **8)** drug elimination from the vitreous via the BRB, **9)** drug elimination from the vitreous via the anterior route. Reproduced with permission from Elsevier [2].

Pharmaceutical treatment of retinal degenerative diseases affecting the posterior segment of the eye is made challenging by restrictive blood ocular barriers such as the blood aqueous barrier (BAB) and the blood retinal barrier (BRB), which separate the eye from systemic circulation [1]. Additionally, the compartmentalized structure of the eye limits the passage of therapeutics from the anterior chamber to the posterior segment, which houses the light-sensing retina [2]. Finally, once the drug successfully enters the back of the eye, effective clearance mechanisms act to rapidly clear the delivered molecules [2]. In conjunction, these barriers render posterior segment ophthalmic drug delivery particularly challenging. Figure 1 provides a schematic representation of the various physical delivery barriers as well as the clearance mechanisms, which effectively expel drugs that successfully enter the eye. 

### Local and systemic routes for drug delivery

It is estimated that following instillation, only 5% of topically applied drugs enter the anterior chamber of the eye, either through trans-corneal permeation (Figure 1, arrow 1) or non-corneal permeation into the anterior uvea through the conjunctiva and sclera (Figure 1, arrow 2) [2]. Increasing the residence time on the eye through viscous formulation can slightly improve uptake. However, due to the physical barrier created by the corneal and conjunctival epithelium, and the relatively small tear volume (~7 μl) available [3], a maximal attainable absorption into the anterior chamber appears to be approximately 10% of the applied dose [4]. Drugs are eliminated from the aqueous humor via aqueous turnover through the Schlemm’s canal and trabecular meshwork (Figure 1, arrow 4) and by uptake into systemic circulation through uveoscleral blood flow (Figure 1, arrow 5) [2]. Elimination via the first route occurs through convective flow at a rate of approximately 3 μl/min and is independent of drug type. Clearance through uveal blood flow however, is influenced by the ability of the drug to penetrate the endothelial walls of the blood vessels. Thus, lipophilic drugs clear more rapidly than hydrophilic drugs, often in the range of 20 – 30 μl/min [2]. Coupled with the physical barrier created by the lens, flow of drugs from the anterior chamber to the posterior segment of the eye is negligible. Therefore, topical drug administration is typically limited to anterior complications. The systemic route is also severely limited in its ability to effectively deliver drugs to the back of the eye. Only an estimated 1 – 2% of compounds delivered via this route successfully cross the BAB (Figure 1, arrow 3) and the BRB (Figure 1, arrow 6) and accumulate within the retinal tissues [1]. With many newly developed pharmaceuticals being protein-based, oral formulations become increasingly difficult to administer, as the drugs need to be protected from degradation within the gastro-intestinal tract. Furthermore, the large concentrations of drug required to achieve therapeutically relevant concentrations within the retinal tissues and the increased potential for off-target interactions makes oral administration an undesirable route of delivery for posterior segment therapies.

**Figure 2:**
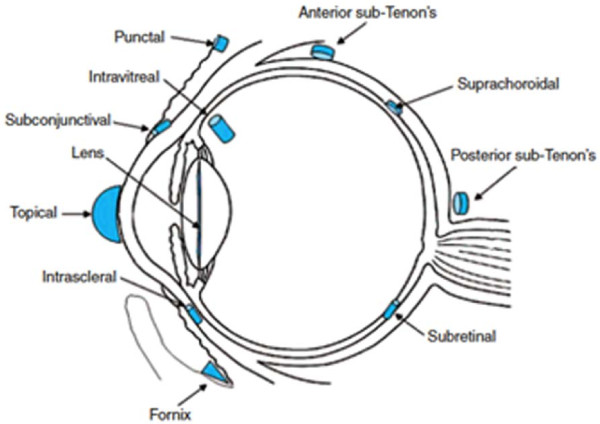
**Potential sites for placement of a drug releasing scaffold in the eye.** An illustration of the numerous potential sites for placement of a drug releasing scaffold for sustained ocular delivery. Reproduced with permission from Nature Publishing Group [3].

There are numerous potential sites surrounding the eye that can house solid drug releasing scaffolds for localized treatment, as illustrated in Figure 2**.**[3]. Periocular instillation that does not require perforation of the eye wall is desirable as it can minimize invasiveness. However, approaches that utilize this route require drugs to pass through several layers, including the episclera, sclera, choroid Bruch’s membrane and retinal pigment epithelium (RPE), in order to reach the vitreous chamber and the retina [5]. Therefore, due to poor penetration into the posterior segment, this route of delivery lacks clinical significance to date [4]. Subconjunctival injections represent an attractive option for delivery of drugs to the choroid as the sclera is highly permeability to large molecules; however, this approach is less appealing for drug delivery to the retina as the compound must still cross the choroid and the RPE [4]. 

### Intravitreal drug delivery

The most efficient means to deliver drugs into the posterior segment is through direct injection into the vitreous cavity (Figure 1, arrow 7) [5]. Using a high-gauge needle, therapeutics may be introduced into the vitreous through simple injection, producing high concentrations of drug locally surrounding the retinal tissues while limiting off-target exposure. However, the concentration of drug is rapidly depleted from the posterior segment via permeation across the BRB (Figure 1, arrow 8) and by diffusion across the vitreous to the anterior chamber (Figure 1, arrow 9), which allows drugs to be cleared through the anterior route [2]. Thus, repeat injections are required, often every 4 – 6 weeks, to maintain therapeutic concentrations of drug within the posterior segment [6]. Repeat instillations are associated with increasing risk of injection-related complications, such as raised intraocular pressure, vitreous or retinal hemorrhage, retinal detachment, retinal tears, endophthalmitis, cataracts, floaters and transient blurry vision [5]. Rates of endophthalmitis and cataract formation per injection are 0.2% and 0.05% respectively [5]. Repeat injections are also associated with patient discomfort and adherence issues [1]. Therefore, while intravitreal injections have the greatest clinical efficacy, they are also the most risky.

Currently, the most promising solutions to combat the challenges of posterior segment drug delivery are approaches that successfully utilize direct intravitreal delivery and sustain therapeutic concentrations for extended periods of time, thereby decreasing the frequency of intervention. The first commercially successful sustained release intravitreal device for treatment of cytomegalovirus retinitis was Vitrasert (Bausch and Lomb), a non-degrading implant that is surgically implanted at the pars plana [5]. Vitrasert is a US Food and Drug Administration (FDA) approved drug delivery system, which consists of a tablet of ganciclovir coated with polyvinyl alcohol (PVA) and ethylene vinyl acetate (EVA) [5]. The impermeable EVA coating limits the surface area through which ganciclovir can release, forcing drug to diffuse through the small PVA rate-limiting membrane, slowing the release and allowing treatment for a period of 5 to 8 months [1]. However, Vitrasert is a relatively large non-degrading device and therefore requires an incision for introduction into the vitreous cavity, as well as a secondary surgical intervention for device removal following exhaustion of the drug reservoir. The I-vation (Surmodics) drug delivery system is another example of a non-degrading, sustained intravitreal release device for the treatment of diabetic macular edema. The helical construct was designed to facilitate ease of implantation and removal, maximize surface area available for drug release, and allow sutureless anchorage within the vitreous [7]. The titanium helix is coated with a blend of poly(methyl methacrylate) and EVA, which is loaded with triamcinolone acetonide and provides sustained release for 18–36 months [5,8]. In contrast, the Iluvien (Alimera Sciences) drug delivery system consists of a very small cylindrical polyimide rod loaded with fluocinolone acetonide (FAc) capable of being injected through a 25-gauge needle and releasing low levels of drug for up to 3 years [5,8]. However, as this scaffold is composed of non-degrading materials and is not fixed to the eye wall, it is expected to remain within the patient’s orbit following depletion of the drug and is currently under review by the FDA [9]. Ozurdex (Allergan), an FDA approved dexamethasone loaded intravitreal insert for the treatment of macular edema and noninfectious uveitis, is another scaffold capable of introduction into the vitreous via minimally invasive injection using a 22-gauge applicator [7]. However, unlike Iluvien, Ozurdex is composed of degradable poly(lactide-co-glycolide) [10], thereby allowing scaffold degradation and clearance from the eye and body without the need for secondary surgical intervention [11].

With recent advances in pharmaceuticals, including regulatory approval of multiple pharmacotherapies to treat wet age-related macular degeneration (AMD), and the increasingly elderly demographic at risk of degenerative eye disorders, there has been renewed interest in designing novel drug delivery platforms, particularly nanocarriers, to address the limitations of posterior segment therapeutics [3]. Furthermore, scientific research is continuing to shed new light on the fundamental biochemical pathways implicated in retinal degenerative diseases, which is leading to the discovery of new pharmacological targets and the development of novel therapeutics.

### RNA interference and siRNA delivery

RNA interference (RNAi) is an evolutionarily conserved mechanism that has been observed in most organisms from plants to vertebrates. It is a mechanism that leads to sequence-specific post-transcriptional gene silencing that was first documented in animals by Andrew Fire and Craig Mello in 1998, both of whom subsequently received the Nobel Prize in Physiology or Medicine in 2006 [12,13].

RNA interference can provide a novel therapeutic modality to treat many human diseases by interfering with disease-causing and disease-promoting genes in a sequence-specific manner. Elbashir *et al.* were the first to demonstrate that small interfering RNA (siRNAs) can induce the RNAi pathway in mammalian cells without producing an adverse immune response [14]. This immediately suggested that the RNAi pathway could potentially be manipulated in humans for the treatment of many human diseases. Theoretically, RNAi can be used to selectively alter the expression of any transcribed gene. This new paradigm in therapeutics allows one to address disease states previously considered ‘undruggable’ [15]. In addition, it creates new opportunities to alter important cellular processes such as cell division and apoptosis, both of which are significantly altered in many cancers [16].

RNA interference is essentially a conserved cellular mechanism that leads to post-transcriptional gene silencing, which can be manipulated for therapeutic applications in humans. Post-transcriptional gene silencing strategies can be broadly divided into four types: 1) single-stranded antisense oligodeoxynucleotides (ODNs)- synthetic molecules that can specifically hybridize with complementary mRNA and sterically inhibit protein translation, 2) ribozymes- catalytically active small RNA molecules that can specifically recognize and cleave single-stranded regions in RNA, 3) microRNA (miRNA)- endogenous, short double-stranded non-coding RNA molecules that play an important role in health and disease by modulating gene expression, and 4) siRNAs- these 18–25 nucleotide long duplexes are potent activators of the innate immune system that have been shown to initiate sequence-specific post-transcriptional gene silencing. Although all of these strategies can potentially be applied to suppress mRNA translation, it is generally accepted that siRNA technology offers the best combination of specificity, potency and versatility as a therapeutic [15]. In addition, siRNAs are easily synthesized and do not require cellular expression systems or complex protein purification systems, making this technology significantly more cost effective over other small molecule therapeutics [12].

Small-interfering RNA mediates its post-transcriptional gene silencing effects via the RNAi pathway. In brief, when exogenous siRNA duplexes are introduced into mammalian cells, the 5’-end is phosphorylated. This duplex is then assembled into a multiprotein complex called RNA-induced silencing complex (RISC), which includes proteins such as Argonaute 2 (AGO2), Dicer, TRBP (HIV-1 TAR RNA-binding protein) and PACT (dsRNA-binding protein) [17]. The sense strand is then cleaved and unwound, leaving only the antisense strand associated with AGO2. Argonaute 2 is an endonuclease that promotes hybridization of this antisense strand to complementary cellular mRNAs and subsequent cleavage of the mRNA target [17]. This results in ‘knocking down’ the translation of the target gene [18].

In designing siRNAs, the three most important attributes to be taken into account are: potency (effectiveness of gene silencing at low siRNA concentrations), specificity (minimize homology to other mRNAs) and nuclease stability (resistance against exonuclease and endonuclease activity). Moreover, there are two types of off-target effects that should be minimized: immune stimulation arising from siRNA recognition by the innate immune system, and unintended silencing of genes that share partial homology with the siRNA [15,17].

It is clear that siRNA technology has a great therapeutic potential in medicine. However, one of the major limitations for their application *in vitro* and *in vivo* is the inability of siRNA to cross cell membranes and reach the cytoplasm. The negative charges arising from the phosphate groups in the siRNA backbone electrostatically repel negatively charged cell membranes, therefore limiting siRNA ability to diffuse across cell membranes. In addition, other challenges common to most drug delivery systems, including high molecular weight, short blood half-life, poor specificity and uptake in target tissues, cellular toxicity, and undesirable off-target effects, significantly hamper the successful application of siRNA therapeutics in medicine [12]. Moreover, the intrinsic physical barriers, efficient drug clearance mechanisms and other complexities of ocular tissues such as the retina and the cornea pose a significant challenge to ocular siRNA delivery. In order to address these problems, several siRNA delivery strategies have been developed for *in vitro* and *in vivo* applications.

Numerous non-viral carriers including natural and synthetic polymers, polyplexes, liposomes, lipoplexes, peptides, dendrimers and free nucleic acid pressurized hydrodynamic injections, as well as virus-based vectors and plasmids encoding for siRNA, have been proposed for siRNA delivery. Although most of these strategies have been attempted with various degrees of success *in vitro* and *in vivo*, strategies for targeted siRNA delivery that are most relevant to ophthalmic applications will be reviewed.

### Non-viral siRNA delivery systems

**Figure 3:**
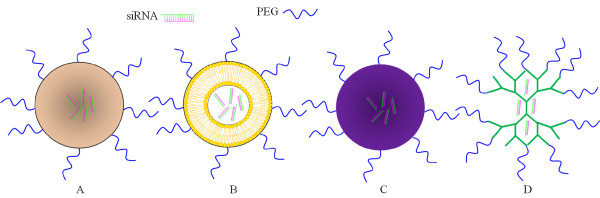
**Nanocarriers for ocular siRNA delivery.** This illustration shows four types of pegylated nanocarriers for ocular siRNA delivery: **A)** polymer, **B)** liposome, **C)** protein, **D)** dendrimer. The siRNA payload is typically entrapped, encapsulated or covalently bound to the nanocarrier interior to preserve its bioactivity, reduce non-specific cellular uptake and prevent undesirable activation of the innate immune system.

In an evolutionary sense, the prevalence of viral infection of cells has likely resulted in highly efficient cellular and systemic defense mechanisms aimed at degrading the naked siRNA molecule *in vivo*. Serum nucleases such as eri-1 [19], renal clearance, and nontargeted biodistribution make intracellular targets extremely difficult to access. Thus, the most prohibitive barrier faced by siRNA therapeutic strategies is a delivery system [20]. Traditionally, engineered viral particles were tasked with the delivery of nucleic payloads to the eye due to its relative immune-privilege status [21]. Several viral types, particularly adenovirus (Ad), adenoassociated virus (AAV), and lentivirus, are being actively investigated as vectors for RNAi therapy [22]. Exotic modifications of these viral vectors, such as self-complementary AAV (scAAV) or helper-dependent adenovirus (HD-Ad), are the current state-of-the-art in viral delivery, optimizing the properties of earlier generations for ocular gene delivery [23]. However, viral vectors are seen as an acceptable rather than perfect solution to nucleic acid delivery; the potential for mutagenesis, limited loading capacities, appropriate targeting, insertional predictability, high production costs, and adverse immune reactivity severely limit the practicability of viruses [24]. Alternatively, delivering plasmid vectors expressing siRNA have been attempted with success [25-27], but such DNA-based expression vectors can potentially integrate into the host genome and increase the chances of insertional mutagenesis [26,28]. Engineered, non-viral siRNA delivery systems are being extensively studied because they are relatively safe and can be easily modified with targeting ligands. These artificial vectors are therefore seen as an attractive alternative for viral delivery systems. There are four main types of vectors that are convenient for non-viral siRNA delivery: 1) polymeric, 2) lipid, 3) protein and 4) dendrimeric nanocarrier delivery systems (Figure 3). 

1) Polymeric nanocarriers

Although many types of polymers have been used to deliver oligonucleotides, much attention has focused on using cationic polymers for two main reasons: 1) their ability to electrostatically bind siRNA without the need for covalent attachment or encapsulation, and 2) the ability of amine containing cationic polymers to provide endosomal buffering and escape for intracytosolic siRNA delivery. Polyethylenimine (PEI) is perhaps the most investigated synthetic cationic polymer for nucleic acid delivery due to its uniquely high buffering capability at endosomal pH, known as the ‘proton sponge’ effect, which releases nucleic acid payloads into the cytoplasm after endocytosis [29]. Grayson *et al.* have demonstrated that polyplexes of PEI can effectively deliver siRNA to cells *in vitro*[30]. Kim *et al.* were among the first to employ the use of pegylated (PEG) PEI-siRNA cationic polyplexes targeted against vascular endothelial growth factor-A (VEGFA), vascular endothelial growth factor receptor-1 (VEGFR1) and/or VEGFR2 to significantly reduce herpes simplex virus-induced angiogenesis and stromal keratitis in murine ocular tissues *in vivo*[31]. Notably, these PEG-PEI-siRNA polyplexes were effective in both local and systemic administration of the formulation. Given that PEI-siRNA has been successfully tested *in vivo* for the treatment of various diseases, it is a promising candidate as a nanocarrier for ocular siRNA delivery [32].

Alternatively, polymeric micelles have been extensively used to deliver nucleic acids. These micelles are colloidal suspensions of amphiphilic copolymers with particle sizes ranging from 5–100 nm [12]. For siRNA delivery, it has been suggested that PEG-polycation diblock copolymers, lactosylated PEG-siRNA and PEG-poly(methacrylic acid) blor siRNA encapsulation are well suited [12]. Interestingly, Duan *et al.* have combined the use of a cationic ock co-polymers fdiblock copolymer (PEI-PEG) with a natural polysaccharide, chitosan, to make ‘ternary’ nanocarriers to successfully deliver siRNA targeted against the IkB kinase subunit mRNA to human Tenon’s capsule fibroblasts *in vitro*[33]. The authors demonstrated that these biodegradable nanocarriers significantly enhanced siRNA delivery and were much less toxic than 25KDa PEI alone. In addition, Ye *et al.* applied these ‘ternary’ siRNA nanocarriers targeting IkB kinase subunit mRNA *in vivo* in a monkey model of glaucoma filtration surgery and showed that subconjunctival injection of these nanocarriers significantly reduced scar tissue compared to controls [34]. Taken together, these results suggest that pegylated cationic nanocarriers may be suitable candidates for ophthalmic siRNA delivery.

2) Lipid nanocarriers

There are many types of lipid-based siRNA delivery systems. However, the most common approaches include: 1) liposomal delivery, where siRNA is encapsulated within vesicles composed of a phospholipid bilayer and 2) lipoplexes, where siRNA complexes with cationic lipids (such as 1,2-dioleoyl-3-trimethylammonium-propane (DOTAP), 1,2-dioleoyl-sn-glycero-3-phosphatidylethanolamine (DOPE), N-[1-(2,3-dioleyloxy)propyl]-N,N,N-trimethylammonium chloride (DOTMA) and N,N-dioleyl-N,N-dimethylammonium chloride (DODAC)) and forms nanoscale complexes. Liposomes are probably the most commonly used artificial gene delivery vector since their ability to transport the preproinsulin gene to the liver was demonstrated nearly 30 years ago [35]. Liu *et al.* have successfully demonstrated that 132 nm pegylated liposome-protamine-hyaluronic acid nanocarriers loaded with siRNA targeted against VEGFR1 can not only enhance VEGFR1 knockdown, but also accelerate intracellular delivery to human RPE cells over free siRNA *in vitro*[36]. After intravitreal administration, these nanocarriers were also able to significantly reduce the area of choroidal neovascularization (CNV) in a laser-induced murine CNV model with minimal toxicity, suggesting their suitability for clinical applications [36]. Lipid combinations such as DC-Chol (3β-*N-*(*N′,N′*-dimethylamino-ethane)carbamoyl]-cholesterol) have also been used to deliver siRNA successfully and may present opportunities to combine desired features to create novel lipid-based nanocarriers [37].

3) Protein nanocarriers

Protein-based siRNA delivery involves the formation of ‘proticles,’ where proteins are conjugated (electrostatically or covalently) to siRNA for delivery. For example, albumin-protamine-oligonucleotide forms nanocarrier complexes (230–320 nm diameter), which can be safely delivered to cells [38]. Recently, Johnson *et al.* have developed a novel cell-penetrating peptide (CPP) for ocular delivery of small and large molecules, including siRNA, fluorescent probes, plasmid DNA and quantum dots to RPE, photoreceptor and ganglion cells *in vitro* and *in vivo*[39]. Not only do the authors report >50% transgene silencing after peptide-siRNA delivery in human embryonic retinal cells *in vitro,* but they also demonstrate that this peptide-based nanocarrier can transduce approximately 85% of the neural retina within 2 h of intravitreal injection *in vivo*[39]. The lack of toxicity, biodegradability and serum stability of these nanocarriers makes them particularly advantageous as a delivery vehicle [38]. However, protein-based nanocarriers have been known to localize and degrade within endolysosomes after cellular uptake [40]. This problem will likely require additional nanocarrier design considerations such as endosomal escape strategies for its successful application in ocular conditions.

4) Dendrimers

Dendrimers represent a group of nanoscale materials that are hyperbranched, monodisperse and have defined molecular weights. Structurally, dendrimers are composed of a central core, repeating units that make up the branches, and surface functional groups [27]. Dendrimers are synthesized in a step-by-step fashion by the sequential addition of repeating units organized in concentric layers, called generations, around the central core. High generation dendrimers have numerous cavities within their hyperbranched structure to allow for the encapsulation of therapeutic agents such as siRNA molecules. The most common dendrimers used for siRNA delivery include poly(amidoamine) (PAMAM) and poly(propylene imine) (PPI) [41]. However, other types of dendrimers composed of amine-containing cationic polymers such as poly-L-lysine have been investigated for ODN (anti-VEGF) delivery to RPE cells *in vitro*[42], and have demonstrated long-term (4–6 months) inhibition (up to 95%) of laser-induced CNV after intravitreal injection in a rat model, without any observable adverse effects [43]. The major advantages of dendrimers include biodegradability, ease of synthesis and customizability, such that they can be synthesized in various sizes and differing number and type of surface functional groups to optimize siRNA delivery. Recently, Agrawal *et al.* have developed dendrimer-conjugated magnetofluorescent nanoworms called ‘dendriworms’ that significantly enhance intracellular siRNA delivery in a mouse model by optimizing endosomal escape [44]. Alternatively, Han *et al.* have conjugated CPPs, such as HIV transactivator of transcription (TaT), to PAMAM dendrimers for enhanced intracellular siRNA delivery *in vitro* and *in vivo*[45]. Together, these results suggest that dendrimers are ideally suited to serve as nanocarriers, which can be loaded with siRNA and functionalized with PEG and targeting ligands for clinical applications. However, at present, there are no examples of dendrimeric siRNA delivery for ocular applications in the literature.

Despite the multitude of siRNA delivery strategies available, the lack of safe and efficient delivery *in vivo* has limited the clinical translation of siRNA therapeutics. Although a few siRNA therapeutic drugs are currently under clinical trials (Table 1, [46-55]) for ocular applications, none have yet been approved by the FDA. Hence, there is a clear need to develop safe and efficacious methods of ocular siRNA delivery. 

**Table 1:** Clinical trials involving siRNA therapeutics for ocular diseases

**Company**	**Drug Name**	**siRNA target**	**Carrier**	**Disease**	**Delivery method**	**Clinical status**	**Reference**
Silence Therapeutics/ Quark/Pfizer	PF-655 (formerly REDD14NP and RTP801i)	RTP801/ DNA-damage- inducible transcript 4 gene (DDIT4)	Naked siRNA	AMD	Intravitreal injection	Phase II – completed	(Pfizer 2011a [50]; Quark Pharmaceuticals 2011a [52]); [55]
Silence Therapeutics/ Quark/Pfizer	PF-655 (formerly REDD14NP and RTP801i)	RTP801/DNA- damage-inducible transcript 4 gene (DDIT4)	Naked siRNA	DME	Intravitreal injection	Phase II – terminated	(Pfizer 2011b [51]; Quark Pharmaceuticals 2011a [52])
Allergan/Sirna	AGN211745 (Sirna-027)	VEGFR1	Naked siRNA	AMD	Intravitreal injection	Phase II- terminated	(Allergan 2008 [46]; Allergan 2009) [47]; [48]
Opko Health	Bevasiranib	VEGF	Naked siRNA	Wet AMD	Intravitreal injection	Phase III-terminated	(OpkoHealth 2011 [49])
Sylentis	SYL040012	ADRB2	Naked siRNA	Glaucoma, Ocular hypertension	Topical	Phase I-completed	(Sylentis 2010 [54])
Quark	QPI-1007	Caspase 2	Naked siRNA	Non-arteritic ischemic optic neuropathy (NAION), Chronic optic nerve atropy, Glaucoma	Intravitreal injection	Phase I – on going	(Quark Pharmaceuticals 2011b [53])

### Chemically modified siRNAs

Various molecular locations on siRNA molecules can be chemically altered to resist hydrolysis and enhance cellular uptake. In order to increase the efficacy of siRNA delivery, much research has focused on increasing the nuclease resistance and therefore serum stability of siRNAs. Nucleases such as eri-1 are involved in the degradation of unmodified siRNA duplexes [19], which have been reported to have a short serum half-life of about 3–5 min. However, it has been shown that siRNA serum half-life can be extended up to 72 h with fully modified duplexes [56].

Among the multitude of possible siRNA modifications, there are two schools of thought regarding the best approach to developing chemically modified siRNA. In one approach, it is believed that extensive chemical modification of siRNA is most likely to lead to the greatest efficacy. For example, Sirna Therapeutics has several patents and products that favour extensive siRNA duplex modifications, where the sense and antisense strands have modified bases (2’-Fluoro-RNA pyrimidines (2’-F-RNA), DNA purines), altered covalent links between the nucleotides (phosphorothioate linkage (PS)) and inverted 5’ and 3’ abasic end caps [17]. These extensive siRNA modifications translated into increased potency and a much longer serum half-life (48–72 h) in a Hepatitis B virus mouse model [56]. In contrast, the other school of thought is focused on creating stabilized siRNAs with minimal modifications. For example, Alnylam Pharmaceuticals has many siRNA products that are selectively modified (2’-sugar modifications such as 2’-O-methyl or 2’-F-RNA) at vulnerable sites, such as those susceptible to endonuclease cleavage [15,17,57]. It is important to note that modifications to the RNA backbone can potentially impair siRNA-induced silencing activity, thus many reported modifications have been limited to the sense strand [58,59]. However, the rules for predicting siRNA stability and potency are still unclear since some studies have demonstrated antisense modifications with preserved siRNA functionality [60,61], while other studies have shown sense strand modifications with reduced siRNA efficiency [62,63].

Various chemical modifications to the terminals, backbone, nucleobases and sugars of siRNAs can be implemented to protect the duplex from exonuclease degradation. For example, the phosphodiester (PO_4_) linkages along the RNA backbone can be replaced with PS or boranophosphonate (PB) at the 3’ end [64-66]. It has been shown that PS derived oligonucleotides stimulate the physical uptake of siRNA in human cells [67], while siRNAs with PB backbone modifications have less cytotoxicity and a much higher nuclease resistance than native siRNA. Such PB siRNAs are at least 10 times more nuclease resistant than unmodified siRNAs, and have recently been used to treat patients with AMD. The process has reached Phase II clinical trials, and it was found to have no observable side effects [68]. Replacement of sugar moieties at the 2’-hydroxyl group of the ribose backbone with 2’-O-methyl, 2’-fluoro, or 2’-methoxyethyl groups can further improve *in vivo* stability [66,69]. Moreover, various molecules can be conjugated to the 5’ or 3’ ends of the sense strand, without affecting the activity of the antisense strand needed for silencing [66]. This method can allow for cell specific targeting or visualization of siRNA uptake and distribution by introducing appropriate ligands and fluorophores respectively. However, degradation of these artificially altered siRNA molecules may result in metabolites with unsafe or otherwise unwanted reactivity [66]. Chemical modification of siRNA can increase stability in biological solutions, target specificity and potency [68]. However, the benefits of modification must be measured against the cost and labour of the modification process, as well as its effects on immune stimulation, which are generally difficult to predict and require empirical testing *in vivo*.

### Immune stimulation and other off-target effects of siRNA delivery

In addition to the gene knockdown effects of the RNAi pathway, there are many other potential consequences that can be initiated by siRNA *in vivo*. Hence, these so called ‘off-target effects’ need to be considered and evaluated in any siRNA delivery study. For example, it is well known that double stranded RNA (dsRNAs) greater than 30 bp are potent activators of the innate immune response [70]. Although siRNA duplexes are shorter than 30 bp, many recent studies have begun reporting off-target effects [58,71]. In general, RNAs are recognized by three major types of immunoreceptors: Toll-like Receptors (TLR), protein kinase R (PKR) and helicases. Toll-like receptors are found on cell-surfaces (TLR3) and in endosomes (TLR3,7,8), whereas PKR and helicases (MDA5, RIG-I) are found in the cytoplasm [72,73]. Immune recognition can lead to a host of downstream effects at the cellular level, including cytokine release, interferon response and changes in gene expression. At the whole body level, the use of unmodified siRNAs have been known to induce systemic toxicity, increase serum transaminases, decrease body weight, lymphopenia and piloerection [74]. Thus, proper siRNA design should likely incorporate features to minimize the possibility of undesirable immune activation.

Although immune activation is influenced by many factors such as oligonucleotide length, sequence, chemical modification, mode of delivery and immune cell type involved, it has been previously shown that chemically modified siRNAs can be synthesized so as to reduce their immunostimulatory properties [74]. However, it is interesting to note that immune stimulation may also have desirable consequences, such as anti-angiogenesis via the TLR3 pathway [72]. Although this type of therapeutic immune stimulation may be useful from the standpoint of treating cancer, it can also have potentially severe side-effects [73].

In addition to immune stimulation, other off-target effects can originate from the partial hybridization of the antisense strand of siRNA with an unintended mRNA. This may lead to the cleavage and subsequent knockdown of the wrong gene [75]. In addition, siRNAs can have their sense strand incorporated into RISC, leading to other off-target effects [75]. To address these problems, siRNA sequences can be carefully selected to minimize complementarity with unwanted mRNAs, and chemically modified siRNAs can be used to increase the selective incorporation of the antisense strand into RISC [62,76]. This highlights the importance of proper siRNA design in mediating target gene knockdown.

### Cellular uptake of nanocarriers and endosomal escape strategies

**Figure 4:**
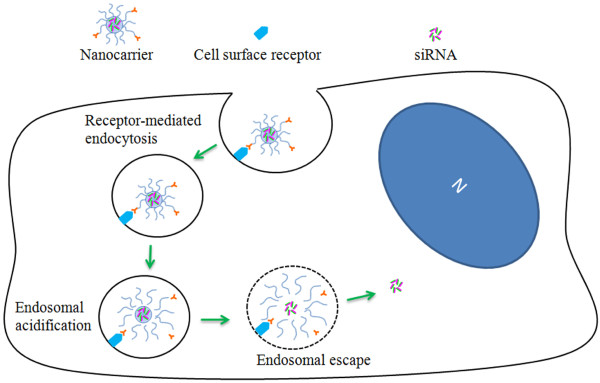
**Nanocarrier uptake and intracellular siRNA delivery.** This illustration shows that uptake of antibody targeted nanocarriers (10–100 nm) occurs via receptor-mediated endocytosis. The key step in cytoplasmic siRNA delivery involves low pH-triggered nanocarrier disassembly and endosomal escape. A ‘smart’ nanocarrier can induce endosomal escape by lysing or fusing with endolysosomes upon acidification. The pH change can also be used to trigger the dissociation of the nanocarrier, therefore releasing the siRNA cargo into the cytosol.

Cells can uptake nanocarriers in many ways, including phagocytosis, macropinocytosis, clathrin-mediated endocytosis, non-clathrin-mediated endocytosis and caveolin-mediated uptake [77]. Each of these pathways delivers nanocarriers to specific cellular compartments, which may help or hinder drugs (intracellular, membrane-impermeable type) from reaching their target site. For example, cationic-lipid-DNA complexes and nanocarriers with ligands for glycoreceptors are internalized via clathrin-mediated endocytosis and are destined for the lysosomal compartment (Figure 4) [77]. In contrast, nanocarriers with ligands such as albumin, folic acids and cholesterol are taken up via caveolin-mediated endocytosis, while cell-penetrating peptide (CPP) ligands such as the HIV transactivator of transcription (TaT), facilitate uptake via macropinocytosis [78,79]. In addition to the surface ligand, the size and shape of nanocarriers can also influence the mechanism and rate of uptake. Previous nanocarrier uptake studies by Rejman *et al.* have shown that untargeted particles up to 200 nm are exclusively internalized via clathrin-mediated endocytosis, while larger particles enter via a caveolin-dependent pathway [80]. Furthermore, they report an inverse correlation between particle size and rate of uptake. For example, as the particle size was raised from 50 nm to 100 nm, internalization was diminished by 3–4 times. Interestingly, their data also suggests that cells have an upper limit for the size of internalized particles, since 1 μm particles were not taken up into mouse melanoma B16 cells *in vitro*[80]. 

After internalization of nanocarriers into cells, many studies have shown that large fractions of these nanocarriers can remain sequestered in trafficking vesicles and endolysosomes [81]. This implies that some types of nanocarriers may not be suitable for delivering membrane-impermeable therapeutics (such as siRNA) to intracellular targets. Moreover, a lysosomal localization of unmodified naked siRNA will likely result in the degradation of siRNA [79]. Hence, much research has focused on intracellular delivery strategies such as cationic lipid transfection, microinjection and electroporation [82]. However, most of these strategies are limited to *in vitro* conditions due to their invasiveness, variable transfection efficiency, complexity of the procedure and potential for altering/disrupting cellular function. Recent efforts have demonstrated that endosomal escape strategies can be incorporated into nanocarrier design to significantly enhance cytosolic delivery of siRNA [83]. Most commonly, CPPs, pH responsive polymers, fusogenic peptide sequences and hydrophobic molecules have been used for nanocarrier endosomal escape [83]. Nanocarriers functionalized with CPPs such as the TaT, VP22, penetratin and polyarginine have been shown to permeate through the plasma membrane for direct cytoplasmic delivery [83-86]. Alternatively, other pH-responsive approaches tend to induce the ‘proton-sponge effect’ for endosomal escape via the clever use of cationic protonable amine-containing polymers such as PEI [87]. In this approach, PEI acts as a buffer against endolysosomal acidification and causes the osmotic swelling and rupture of endolysosomes, releasing the nanocarriers into the cytosol (Figure 4) [88]. In contrast, other approaches attempt to conjugate drugs to fusogenic peptide sequences, such as GALA and KALA, or hydrophobic molecules such that the nanocarrier can traverse membranes [83]. For example, cholesterol-tagging has been shown to improve cytosolic delivery of siRNA with minimal cytotoxicity [89].

Interestingly, lipid-based nanocarriers can also be engineered to fuse with cell membranes, either avoiding endocytosis completely or escaping endolysosomes without inducing endolysosomal lysis. Although some studies suggest that a net positive surface charge and a high cationic lipid/siRNA molar charge ratio are important factors required to facilitate efficient membrane fusion with lipid-based nanocarriers, it has been reported that these factors also seem to significantly increase toxicity [90]. Recently, Leal *et al.* have reported the development of cationic liposome (CL)-siRNA complexes with novel cubic phase nanostructures, which offer a novel solution to lipid based delivery. Cubic phase lipid delivery systems readily fuse with cell membranes due to their high charge density and positive Gaussian modulus, delivering their cargo through transiently induced pores in the endosomal membrane, which results in highly efficient gene silencing *in vitro* with low toxicity [91,92]. In contrast, some studies have successfully employed non-invasive physical methods to enhance intracellular delivery of siRNA. For example, Du *et al.* recently demonstrated that simultaneous administration of low intensity ultrasound or 15-20% microbubbles can safely enhance the delivery efficiency of siRNA-loaded polymeric nanocarriers to rat RPE-J cells *in vitro*[93]. It is likely that a combination of approaches will need to be tested to determine the optimal strategy for endosomal escape for ocular siRNA delivery.

### Development of a ‘universal’ nanocarrier for ocular siRNA delivery

To achieve intracellular ocular siRNA delivery via intravitreal injection, a rational design of a nanocarrier is required that is capable of overcoming the unique biological barriers present in the eye. A review of the literature suggests that several important features including targeting, stealth, siRNA incorporation, size, shape and surface characteristics will have to be taken into consideration for the development of a ‘universal’ nanocarrier for ocular siRNA delivery (Table 2). Turchinovich *et al.* recently demonstrated efficient siRNA delivery into mouse retina *in vivo* using a commercially available transfection reagent [94]. However, this non-targeted method mainly transfected the retinal ganglion cell layer. This suggests that it is likely necessary to use targeting molecules on nanocarriers to control the specific retinal cell type being targeted for transfection. Other studies by Aggarwal *et al.* have shown that nanocarriers exposed to biological fluids in host tissues such as serum are immediately coated with opsonins and other host proteins, creating a ‘molecular signature’ that determines the internalization pathway and fate of nanocarriers taken up by phagocytic cells [95]. Given these observations, many approaches to shield the nanocarriers from such host-induced modification have been developed, among which, a hydrophilic coat of PEG has demonstrated its effectiveness *in vivo*. These PEG coated ‘stealth’ nanocarriers have been shown to significantly reduce non-specific cellular uptake and opsonization by phagocytic cells [96]. 

**Table 2:** Literature review of ocular siRNA nanocarrier delivery

**Target**	**Carrier**	**Disease**	**Model**	**Delivery method**	**Results**	**Implications for ocular diseases**	**Reference**
IκB kinase beta (IKKβ)	Cationic nano-copolymers CS-g-(PEI-b-mPEG)	Glaucoma filtration surgery	Rhesus monkey	Subconjunctival injection	Marked reduction in subconjuctival scarring with siRNA treatment in monkeys with trabeculectomy; higher blebs with siRNA compared to PBS treatment; less fibrosis and less destruction of local tissue in siRNA-treated eyes	Improved surgical outcome in glaucoma filtration surgery (less scarring)	[34]
IκB kinase beta (IKKβ)	Cationic nano-copolymers CS-g-(PEI-b-mPEG)	Glaucoma filtration surgery	Human	*In vitro* transfection	Downregulation of IKKβ at the mRNA and protein levels; nuclear factor-κB (NF-κB) inhibited in human Tenon’s capsule fibroblasts	Decreased scar formation following glaucoma filtration surgery	[33]
VEGFR1	PEGylated liposome- protamine- hyaluronic acid nanoparticles (PEG-LPH-NP)	Choroidal neo-vascularization	Human RPE cells (ARPE19) and rats	Intravitreal injection	Reduced laser-induced CNV area in rats by PEG-LPH-NP-S nanoparticles (anti-VEGFR1 siRNA) compared with naked siRNA and PEG-LPH-NP (negative siRNA); downregulated VEGFR1 expression in human RPE cells with siRNA compared to naked siRNA and control group; no significant retinal toxicity	Delivery of siRNA to decrease CNV with low toxicity	[36]
Non-specific commercial siRNA	Transit- TKO transfection reagent	Healthy mice	Mouse	Intravitreal injection	Combination of siRNA with Transit - TKO transfection reagent penetrated through the inner limiting membrane into the retina and accumulated in ganglion cell layer	Uniform delivery to retinal through intravitreal injections of siRNA using commercial reagents	[94]

Moreover, the use of a nanocarrier allows for the control of immune stimulation. Kleinman *et al.* have shown that siRNA can directly mediate CNV suppression *in vivo* via a non-RNAi mediated mechanism involving cell-surface receptor TLR-3 [72]. A therapeutic siRNA shielded from the ocular environment can perhaps avoid such immune stimulation effects of siRNA. However, in some cases, it might be desirable to induce a potentially beneficial immune stimulation effect such as angiogenesis suppression. Given the versatility of nanocarrier systems, it is likely possible to design a carrier that exposes chemically modified, stabilized siRNA to ocular fluids to mediate innate immune stimulation and trigger the TLR-3 pathway for angiogenesis suppression.

**Figure 5:**
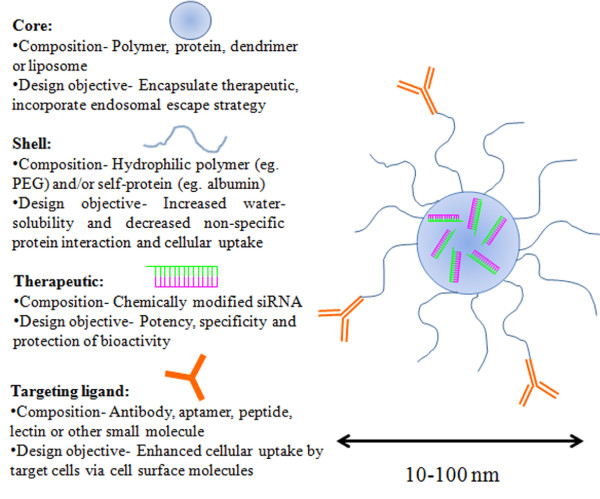
**Schematic of a four-component ‘universal’ nanocarrier for ocular siRNA delivery.** This illustration highlights the salient features of a four-component, targeted core-shell nanocarrier for ocular siRNA delivery.

A review of successful siRNA delivery nanocarriers *in vivo* strongly suggests that a four component core-shell delivery system is ideal: 1) core- composed of a biodegradable material that entraps, encapsulates or covalently binds siRNA, 2) shell- composed of a hydrophilic polymer such as PEG or a self-protein such as albumin for stability, protection and surface charge modification, 3) drug- chemically modified siRNA for enhanced stability, potency, specificity and efficacy, 4) targeting ligand- antibody, aptamer, peptide, lectin or other small molecules present on the nanocarrier surface for selective delivery to target cells (Figure 5). In addition, the size, shape and surface characteristics of the nanocarrier are key elements that control their biological interactions. Although the ideal size and shape of nanocarriers for ocular drug delivery have not been systematically tested, the diffusion of nanocarriers through solid tumor models suggest that smaller carriers are preferred over larger ones. Wong *et al.* have recently provided proof-of-principle that gelatin nanocarriers can be designed to change their particle size from 100 nm to 10 nm upon reaching the tumor microenvironment, responding to locally produced matrix metalloproteinase-2 (MMP-2), and can thus penetrate deeper into the tumor tissue [97]. Although most studies involving nanocarrier biodistribution and cellular uptake have been elucidated using spherical nanocarriers, recent studies suggest that the shape of nanocarriers can significantly influence their biological interactions [79,98]. Particularly, a recent study showed that positively charged cylindrical particles with an aspect ratio of 3 (150 nm x 450 nm) were internalized four times more rapidly by HeLa cells than cylindrical particles with an aspect ratio of 1 (200 nm x 200 nm) [98]. This suggests that it is important to consider the size as well as the shape of the nanocarrier in their design. Nanocarrier biodistribution and uptake in biological systems can also be controlled by manipulating their surface characteristics. The predominant strategy for improving the stability of nanocarriers in biological solutions has involved the grafting of PEG to the surface to render them more hydrophilic and neutral in charge [79]. Some studies suggest that the addition of self-proteins such as albumin via adsorption or covalent modification may reduce non-specific cellular uptake and opsonization [99,100]. Taken together, these data suggest that it is important to optimize the size, shape and surface characteristics for the development of a ‘universal’ nanocarrier for ocular siRNA delivery. 

The proposed four-component nanocarrier system provides a customizable platform for the development of a ‘smart’ drug delivery system that can be engineered to enhance endosomal escape, control siRNA release intracellularly and manipulate the innate immune response. Particularly, a core-shell nanocarrier structure allows for the incorporation of specific endosomal escape strategies, which can be activated upon endocytosis. For example, the core and shell components can be joined with a cleavable linker that is sensitive to endolysosomal stimuli such as acidic pH and acid-activated proteases. This design effectively allows for the de-shielding of the nanocarrier core, containing siRNA, to induce endosomal destabilization, or to directly traverse the endosomal membrane if the core has a hydrophobic composition. The reducing environment of the cytosol can also be used to further stimulate the dissociation of siRNA from the nanocarrier core via the incorporation of disulphide bonds. The first successful systemic delivery of siRNA via a targeted nanocarrier in humans serves to confirm these important parameters in nanocarrier design [101].

### Conclusions and future directions

Given that we currently lack an ideal siRNA delivery system for ocular disorders, it is instructive to consider the nucleotide delivery strategies found in nature. For example, viruses are essentially targeted biological nanocarriers for the local or systemic delivery of nucleic acids, known to be the causative agents of various human diseases. A virion is indeed a smart nanocarrier, with several key features: environmental stability, monodispersity, bioresponsiveness, biodegradability, immune modulation properties, endosomal escape capabilities, intracellular replicative capacity, and targeted and localized DNA/RNA intracellular delivery to specific cells for controlling gene expression. To this extent, Breitbach *et al.* have recently shown that a modified oncolytic pox virus administered intravenously in human subjects can selectively target cancer cells in solid tumors, without any observable clinical effects on normal cells [102]. This 300 nm enveloped virus delivered ds-DNA to target cells in a dose-dependent manner, similar to that observed in the recent Phase I clinical trial with siRNA-nanocarrier technology [101]. A nature-inspired nanocarrier design can potentially provide structural insights into developing the optimal solutions to some of the major barriers in ocular and systemic siRNA delivery.

Many groups have employed ‘smart’ nanocarriers or ‘synthetic viruses’ that mimic isolated aspects of viral nucleotide delivery with varying degrees of success. For example, Hu *et al.* developed a pH-responsive core-shell nanocarrier designed to release various cargos including proteins, viral particles and siRNA under endosomal acidification [103]. However, most of these single-stimuli responsive nanocarriers are focused on either drug delivery or for diagnostic purposes (imaging and detection), without the ability to combine such useful features. Although multiple stimuli-responsive nucleotide delivery systems are currently under development to address this challenge, a general strategy for intracellular nucleotide delivery has not yet been established [104]. This may be due to the fact that nucleotide delivery systems vary greatly in their composition, such that combining beneficial features of two different nucleotide delivery systems into a hybrid system may not always be possible. In designing a nucleotide delivery system, it is instructive to note that viruses sequentially deploy specific strategies to overcome each barrier at the tissue and cellular level for successful intracellular nucleotide delivery. It follows that any clinically viable nucleotide delivery system will have to take into account the common barriers to siRNA delivery and incorporate specific strategies to overcome each of these barriers, while being flexible enough to combine features that can be adapted to several ocular conditions.

 We envision that the ultimate ocular siRNA delivery system would incorporate a combination of nature-inspired desirable features: a biodegradable, multiple stimuli-responsive nanocarrier for controlled and localized siRNA release targeted to specific cell types for manipulating gene expression of specific genes. When combined with a drug delivery device, such a ‘smart’ nucleotide delivery system would not only address the current challenges of ocular siRNA delivery, with improved biodistribution, bioavailability and reduced toxicity, but also improve therapeutic outcomes for the patient.

## **Abbreviations**

AAV: Adenoassociated virus; Ad: Adenovirus; AMD: Age-related macular degeneration; AGO2: Argonaute 2; BAB: Blood aqueous barrier; BRB: Blood retinal barrier; CNV: Choroidal neovascularization; CPP: Cell-penetrating peptide; DC-Chol: (3β-[N-(N′,N′-dimethylamino-ethane)carbamoyl]-cholesterol; DODAC: N,N-dioleyl-N,N-dimethylammonium chloride; DOPE: 1,2-dioleoyl-sn-glycero-3-phosphatidylethanolamine; DOTAP: 1,2-dioleoyl-3-trimethylammonium-propane; DOTMA: N-[1-(2,3-dioleyloxy)propyl]-N,N,N-trimethylammonium chloride; dsRNAs: Double stranded RNAs; EVA: Ethylene vinyl acetate; FAc: Fluocinolone acetonide; FDA: US Food and Drug Administration; 2’-F-RNA: 2’-Fluoro-RNA; HD-Ad: Helper-dependent adenovirus; miRNA: microRNA; MMP-2: Matrix metalloproteinase-2; ODNs: Oligodeoxynucleotides; PACT: dsRNA-binding protein; PAMAM: Poly(amidoamine); PB: Boranophosphonate; PEG: Polyethylene glycol; PEI: Polyethylenimine; PKR: Protein kinase R; PO4: Phosphodiester; PPI: Poly(propylene imine); PS: Phosphorothioate linkage; PVA: Polyvinyl alcohol; RISC: RNA-induced silencing complex; RNAi: RNA interference; RPE: Retinal pigment epithelium; scAAV: Self-complementary AAV; siRNA: Small interfering RNA; TaT: HIV transactivator of transcription; TLR: Toll-like Receptors; TRBP: HIV-1 TAR RNA-binding protein; VEGFA: Vascular endothelial growth factor-A; VEGFR1: Vascular endothelial growth factor receptor-1.

## Competing interests

No competing interests to declare.

## Authors’ contributions

AT, SF, AZ, KK and BM contributed towards writing and editing the manuscript. GH and HS critically evaluated the manuscript for publication. All authors read and approved the final manuscript.

## Authors’ information

No information to share.
